# Encouraging Emotional Conversations in Children With Complex Communication Needs: An Observational Case Study

**DOI:** 10.3389/fpsyg.2021.674755

**Published:** 2021-07-06

**Authors:** Gabriela A. Rangel-Rodríguez, Mar Badia, Sílvia Blanch

**Affiliations:** Department of Basic, Developmental and Educational Psychology, Universitat Autònoma de Barcelona, Barcelona, Spain

**Keywords:** emotion, complex communication needs, augmentative and alternative communication, interactive learning environments, emotional education, family, parent–child interaction, home reading

## Abstract

Children with complex communication needs (CCN) regularly have barriers to express and discuss emotions, and have fewer opportunities to participate in emotional conversations. The study explores and analyzes the changes after a training program focused on offering an interactive home learning environment that encouraged and modeled emotion-related conversations between a parent and a child with CCN within storybook-reading contexts. An observational design (nomothetic/follow-up/multidimensional) was used to explore and analyze the changes in the communicative interaction around emotions between mother-child. Augmentative and alternative communication (AAC) technologies were used to provide the child access to emotion-related vocabulary. The training program resulted in the mother providing more opportunities to engage her child in emotional conversations, suggesting that when opportunities and resources to talk about emotions were promoted, the child showed more engagement in emotion-related conversations using his AAC system. The mother–child communicative patterns and behavioral relationships observed during the phases are also presented. This case study illustrates the importance of a primary communication partners’ role in facilitating emotional conversations, and the promising efficacy of a training program implemented in a storybook interactive learning environment to promote conversations about emotion-related events while encouraging children with CCN to learn, explore, express, and discuss emotions.

## Introduction

Communication and language are essential to understand, express, and adaptively regulate and respond to emotions. Children with complex communication needs (CNN) may have impairments in language production and/or comprehension (Beukelman and Light, 2020), resulting from different etiologies such as cerebral palsy, Down syndrome, developmental disabilities, or speech-language impairment. Recent studies have revealed some evidence that people with CCN often face barriers in expressing and/or understanding emotions, and may have fewer opportunities to talk and learn about emotions (Na and Wilkinson, 2018; Rangel-Rodríguez et al., 2021; Wilkinson et al., 2021).

The literature has shown that learning to express and communicate emotions linguistically and appropriately (according to socio-cultural and family norms) is related to adaptive emotional-behavioral outcomes. As an illustration, individuals who are able to express their emotions linguistically (e.g., emotional vocabulary) are more likely to be aware of and recognize their own and others’ emotions, exhibit less intense and sustained emotions, present more emotional management strategies, and display effective ways to self-advocate (Test et al., 2005; Cole et al., 2010; Roben et al., 2013; Doyle and Lindquist, 2018; Torre and Lieberman, 2018). Children with CCN often present some challenges in expressing emotions, not only via linguistic modes of communication (e.g., difficult to produce speech or to access vocabulary that enables them to understand and express emotions), but also in non-linguistic modalities (e.g., motor and/or sensory difficulties). Thus, their communication partners may face difficulties in identifying, interpreting, and discussing emotions (Wilkinson et al., 2021) with a child and may over or underestimate the child’s emotional experience (Reed et al., 2020). As a result, emotional learning for children with CCN can be challenging, restricted, or even ignored.

There is a significant body of evidence on the benefits of augmentative and alternative communication (AAC) in supporting the language and communicative development of children with CCN (Light and McNaughton, 2012). To have effective communication through AAC, it is critical to offer interactive and dialogic learning environments that support those who rely on AAC and their communication partners (Dattilo and Camarata, 1991; Kent-Walsh and McNaughton, 2005; Ogletree et al., 2016; Beukelman and Light, 2020). Dialogic and interactive learning environments must be created to maximize children’s learning opportunities and outcomes (García-Carrión et al., 2018). In addition, encouraging conversations between children and communication partners provides opportunities to interact, express, and share thoughts, opinions, emotions, knowledge, as well as create new learning (Vygotsky, 1962; Rogoff, 1990; Brinton and Fujiki, 2011). Interactive learning environments that promote conversations are also beneficial for supporting social, emotional, and communicative learning outcomes for children with special needs (Gottman et al., 1997; Jenkins et al., 2003; Schmidt and Stichter, 2012; Fleury and Schwartz, 2017). However, research on the possible benefits of AAC strategies to support children’s emotional development remains scarce (Wilkinson et al., 2021) and “desperately needs direct attention” (Na et al., 2016, p. 447).

Emotion talk refers to having conversations about emotion-related events. Emotional conversations are a medium to foster emotional learning, which means that children must understand words that describe emotions and also have access to emotion-related vocabulary. Through dialogue, communication partners can discuss and teach the language of emotions, and they can suggest strategies for managing and understanding emotional experiences (Eisenberg et al., 1998; Eisenberg and Morris, 2003; Morris et al., 2007; Tenenbaum et al., 2008; Aznar and Tenenbaum, 2013; Harris et al., 2018). Hence, children can learn skills such as recognizing and labeling emotions, comprehending their causes and consequences, talking about them, and choosing appropriate ways to manage and respond to different emotions they and their partners experience (Saarni, 1999; Saarni et al., 2007; Beck et al., 2012). Suggestions have been proposed to design interventions that promote opportunities to have conversations about emotions with children who could benefit from AAC. Na et al. (2016) suggested that initially, communication about emotions should occur during an enjoyable and meaningful activity with the child (e.g., storybooks, videos, movies, games, role-playing, TV programs, morning conversations, etc.). Initiating emotional discussions amid a heightened emotional state (e.g., in the course of a temper-tantrum) is not ideal (Wilkinson et al., 2021). These assumptions are consistent with studies that indicate the importance of presenting a joyful and comfortable context in teaching practices and its positive relationship in facilitating students’ learning (Schutz and Lanehart, 2002; Willis, 2007; Bueno and Forés, 2018).

Another key aspect for an effective interactive learning environment that supports the development of children with or without speech, language, or communication needs is the skills and performance of communication partners (Brinton and Fujiki, 2011; Romski et al., 2011; Kent-Walsh et al., 2015; Mermelshtine, 2017; Biggs et al., 2018; O’Neill et al., 2018). Partners must learn scaffolding strategies such as providing opportunities to talk and learn about emotions (making comments, asking questions, etc.), modeling the use of a child’s communication system, and offering feedback (Na and Wilkinson, 2018; Wilkinson et al., 2021). Brinton and Fujiki (2011) illustrated the crucial role of communication partners’ attitudes in children’s development by pointing out that “emotion talk that is carefully constructed and timed to be most accessible to children can support the development of both emotional competence and social communication” (p. 271). The role of communication partners is essential to support children’s socio-emotional and communicative learning.

Additionally, to promote conversations about emotions in children with CCN, it is essential to design AAC systems that provide significant emotional vocabulary and a diverse range of emotion-related communication tools that are culturally sensitive to the child and the family’s linguistic and cultural context (Blackstone and Wilkins, 2009; Na et al., 2016; Wilkinson et al., 2021). For example, vocabulary that serves to explain why a person feels the way they feel (e.g., “I’m irritated because it’s too noisy”), and some possible responses to those emotions (e.g., “I need a break and go somewhere else”). The AAC system must be functional for the child to communicate about emotions and useful for the child’s partners to model emotional communication. Interviews can be critical to gather the information that guides intervention decisions that support communication about emotions. The Early Development of Emotional Competence (EDEC) is a semi-structured interview developed to meet this purpose (Na et al., 2018).

Evidence concerning the use of AAC systems in conversations about emotions provides some insight about the promising benefits of supporting children who have CCN. Na and Wilkinson (2018) developed the Strategies for Talking about Emotions as PartnerS (STEPS) program and examined it with three parents and their children with Down Syndrome, conducting a single-subject multiple-baseline across participants design. They selected storybook time as the context to foster conversations about emotions. Interactive storybook reading has the advantage of involving the child in an active role and provides a rich and natural setting for emotional and language development (Bedrosian, 1999; Drummond et al., 2014; LaForge et al., 2018). The STEPS program focused on supporting communication partners to implement communicative strategies for encouraging conversations about emotions with children with CCN. The STEPS training (see Na, 2015; Na and Wilkinson, 2018; Wilkinson et al., 2021) consists of three steps: Step 1: provide and model emotional vocabulary (label the emotion); Step 2: validate and discuss emotions (talk about the reason for the emotion); Step 3: communicate about appropriate responses to emotions (talk about the possible responses/coping strategies to emotion). These steps, in combination with other communication partner strategies (e.g., ask, wait, provide feedback) and the design of emotional-communication boards resulted in parents providing more opportunities to discuss emotions using the AAC system, and the children increasing their utterances referring to emotions using different communication modes (including AAC). Even though further research is needed, the STEPS program appears to be a beneficial resource to initiate conversations about emotions with children who have CCN in natural settings.

Giving children with CCN access to key and meaningful emotion-related vocabulary, as well as encouraging its usage, is critical to support effective conversations between the children and their communication partners (Rangel-Rodríguez et al., 2021; Wilkinson et al., 2021). Nuclear family members are life partners and the primary communication partners in the child’s social networks (Blackstone and Hunt-Berg, 2012); its engagement in children’s healthy development, learning, and emotional well-being are fundamental (Mandak et al., 2017; Lehrl et al., 2020). Therefore, families are certainly “children’s first and most important teachers, advocates, and nurturers” (U.S. Department of Health and Education and Human Services and U.S. Department of Education, 2016, p. 1). Supporting families in promoting learning environments is essential to aid children’s learning (Lehrl et al., 2020) and thus reduce or prevent behavioral, social, or emotional conflicts in the future (Sanders, 2008; Dishion et al., 2014).

The current study describes and analyzes a program designed to increase and encourage conversations about emotions during a storybook reading activity with a child who has limited speech using a case study approach. The goal of the program is to facilitate interaction skills that encourage emotional talk. This study is part of a larger research project carried out by the first author.

## Materials and Methods

### Design

This study highlights the importance of the sociocultural context, communication, language, and experiences generated from the intervention, as well as examining the efficacy of the program through the behaviors of its participants in natural settings. Therefore, a paradigm that allows an integrative and complementary study was needed. A pragmatic epistemological framework and mixed-method approach were used to allow for the coexistence, integration, and/or combination of quantitative and qualitative elements in the study and enable the use of analytical techniques in either sequential or parallel phases (Teddlie and Tashakkori, 2010; Anguera et al., 2020). For the present study, an observational methodology was employed.

This case study was carried out by an observational design, which was configured based on three dichotomous criteria (Anguera and Izquierdo, 2006):

• Unit of study: one unit or individual (*idiographic*) or a group of units/participants (*nomothetic*) studied.
• Temporality: one session (*point*) or several sessions (*follow-up*) observed over time.
• Number of dimensions: one (*unidimensional*) or several (*multidimensional*) behaviors considered to study.

Hence, this study employed a nomothetic/follow-up/multidimensional observational design (N/F/M) for the following reasons:

• Nomothetic: a parallel and independent analysis of the behavior of the child and the mother was conducted.
• Follow-up: intra and inter-sessional recordings analyses between the 4 phases of the program (13 complete storybook reading sessions) from the collected data were performed.
• Multidimensional: several dimensions of interactive responses from the child and the adult in each session were recorded.

The observation was direct through video recordings of storybook reading sessions that the mother shared with the researcher, allowing the researcher full auditory and visual accessibility of the interaction.

### Participants

A mother–child dyad participated in this study. They were recruited through convenience sampling. The inclusion criteria were (1) have a child who has functional hearing and vision per parent report and CCN, with previous or current exposure to aided AAC systems; (2) parents who have no speech, language, or hearing impairments; and (3) have an internet connection. The mother participated in the study and although the father could not participate, he was also interested in the study.

The mother was 44 years old, and the child was 7 years old. The child had a medical diagnosis of dyskinetic and dystonic cerebral palsy that affected the ability to control muscle movement, posture, and coordination; specifically, characterized by slow unintentional writhing movements (dyskinetic) and varying patterns of muscle tone (dystonia) (Center for Disease Control and Prevention (CDC), 2020). The child’s speech intelligibility was severely impaired. The mother stated that her child “understands everything and can sound most of the words, but the sound level is very faint (lots of air in the sound),” so he also communicates via gestures (e.g., eyes up for yes, eyes down or stick his tongue out for no), facial expressions, and through an AAC electronic device. The child has used a speech-generating device since he was 2 years old. Currently, he accesses his device through eye-gaze. The mother commented that he has functional hearing and vision level, uses glasses, and attends a 1st grade class in a mainstream school program.

Ethical approval was obtained from the Ethics Committee from the Autonomous University of Barcelona prior to starting the research.

### Materials

Storybooks were selected for the program, specific materials were used to illustrate the training session conducted with the mother, and communication boards were designed in conjunction with the mother to provide the child access to emotion-related vocabulary.

#### Storybooks

The selected storybooks had to fulfill the following criteria: (a) being an illustrated book, (b) with text appropriate to the child’s characteristics, interests, and cultural background, (c) showing at least two different emotional categories (e.g., sad–happy), and (d) a length of at least 20 pages. The selection criteria taken was proposed by Na and Wilkinson (2018), who adapted the guidelines from Kent-Walsh et al. (2010).

#### Instruction Session Materials

##### STEPS instruction page

The STEPS instruction page contains a detailed description of each step proposed in the training to encourage communication about emotions during the storybook-reading activity (see Supplementary Material 1). The mother received a copy of the instruction page as support. This page was an adaptation of the handouts suggested by Na and Wilkinson (2018; Wilkinson et al., 2021), where suggestions to encourage mother–child communication were included.

##### Communication board design page

To create communication boards that were culturally and family appropriate, the researcher asked the mother to read the selected books and (a) choose the emotions with which she could feel comfortable talking with her son; (b) identify the causes for the emotion; and (c) propose possible responses or coping strategies to these emotions. The communication board design page contained a table to write the book page, the emotion selected to talk about, the trigger for that emotion, and the possible responses when that emotion appears.

##### Communication board example

According to the child’s AAC communication, the clinician designed an example of pages to explain how the boards could be created in the child’s current AAC system (see Supplementary Material 2).

##### Video-demonstrations of storybook reading activity

Five short videos, around 1 min each, were presented to the mother in the training session. Each video showed a role-playing situation between two individuals (one acting like a child and the other as a parent) in a storybook reading activity. Each video explained the different parts of the training.

#### Communication Boards

**FIGURE 1:**
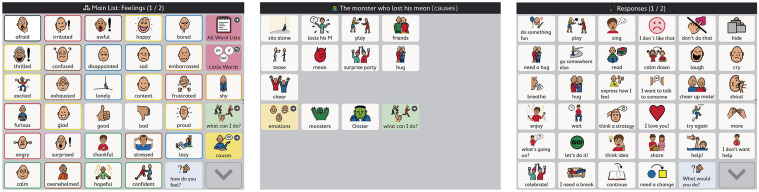
Example of emotion communication dynamic display page [emotions, causes, and responses to emotions]. The Picture Communication Symbol^®^ and Boardmaker by Tobii Dynavox^®^ All Rights Reserved. Used with permission.

Once the mother filled in the material “communication board design page” for each book, suggestions were made by the researcher and agreed upon by the mother. The researcher then created the communication board pages using the child’s communication system (Snap-Core First App, a system that permits dynamic display pages), and shared it with the mother. Vocabulary was added to the emotion page in his AAC system, as needed. Also, new communication pages were created throughout the program: (a) one page per book to talk about the possible causes of the emotion, and (b) one section with vocabulary to talk about possible responses to emotions. As the mother suggested new words that enabled her and the child to discuss emotions, the child’s access to emotion vocabulary grew. An example of the child’s AAC emotion-related pages used is presented in Figure 1.

### Instruments

#### Observation Instrument

To analyze the data collected in an interactive natural context between communication partners and children with CCN, an observation tool was constructed *ad hoc* to fully adapt to the interests of the research (Anguera et al., 2021), based on the data obtained from preliminary interactions observed (15 mother–child with CCN dyads in a storybook-reading activity), and previous theoretical and empirical work (Girolametto et al., 2007; Kent-Walsh et al., 2010; Girolametto and Weitzman, 2011; Rowland, 2011; Parish-Morris et al., 2013; Poyatos, 2015; Na and Wilkinson, 2018). Therefore, the instrument combined a field format with category systems: “this combination is possible when some or all of the dimensions in the field format have a theoretical framework and the object of research is atemporal” (Anguera et al., 2018, p. 7).

**FIGURE 2:**
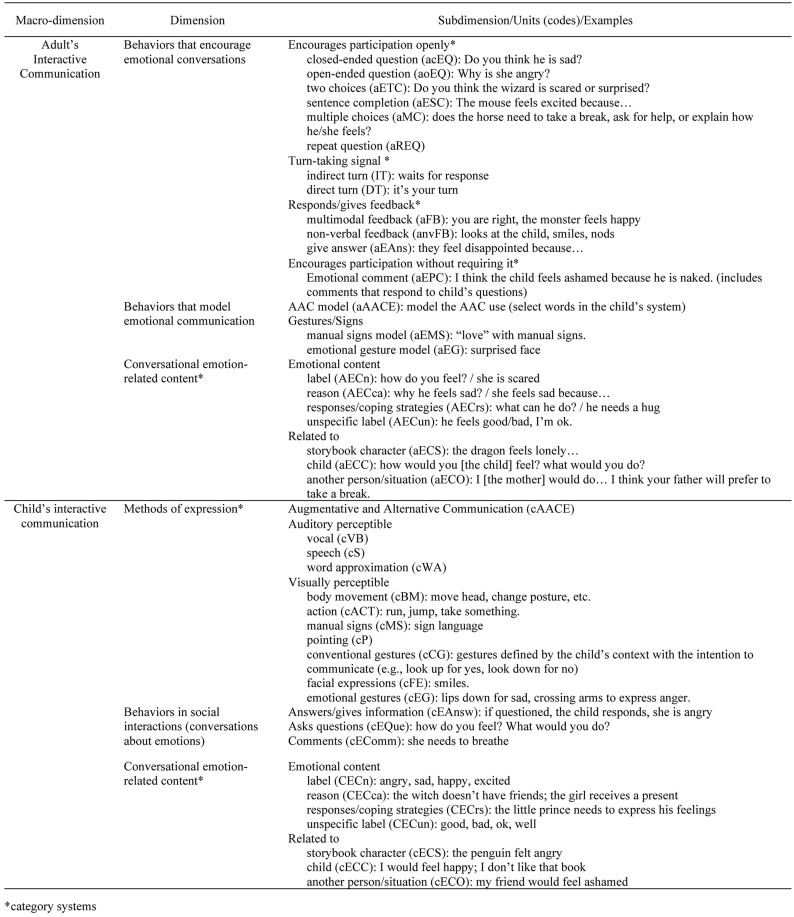
Observation instrument.

The full-version observation instrument is presented as Supplementary Material 3. Figure 2 only presents the dimensions and units analyzed for the purposes of the present study, which includes 6 dimensions (out of the 15 dimensions included in the full-version) that allowed for the capturing of mother-child emotional interaction in the storybook reading activity.

#### Recording and Analysis Instruments

All the video-recording sessions were recorded and coded according to the full-version observation instrument using the software LINCE 1.4 (Gabin et al., 2012). The data obtained were time-based and concurrent, categorized as type IV; that is, “the observer notes the duration of events, but different events can overlap and occur together” (Bakeman, 1978, p. 65).

For data analysis, different software were employed: GSEQ 5.1 (Bakeman and Quera, 2011) to conduct the intra-observer reliability and lag-sequential analysis, HOISAN 1.6.3.3.6 (Hernández-Mendo et al., 2012) for the polar coordinate analysis, and the R program to obtain the graphic representation on polar coordinate analysis (Rodríguez-Medina et al., 2019).

### Procedures

The study procedures consisted of an interview and exploratory observations of a video-recorded storybook reading activity at home, followed by a training session and post-observations of videorecords of the storybook interactive learning environment suggested during the program.

Once the mother and child were selected for participation, a semi-structured interview called EDEC, the Early Development of Emotional Competence tool (Na et al., 2018), was conducted between the researcher (the first author) and the mother. The purpose of the interview was to identify the current child’s emotional state and communicative characteristics, as well as some caregivers’ emotional and communicative socio-cultural aspects, in order to support the family better (Rangel-Rodríguez et al., 2021). In this meeting, the researcher explained the study and the importance of recording the storybook reading sessions throughout their participation. The mother provided informed consent and agreed to participate in the study.

As part of the interview, the mother was asked to share her son’s favorite storybooks. As the sessions progressed, other storybooks were suggested based on the research’s criteria, family values, and the child’s interests and preferences.

The training program was an adaptation of Na and Wilkinson’s (2018) protocol and consisted of four phases [(1) exploratory, (2) strategy implementation, (3) iteration, and (4) maintenance]. One additional training session was carried out after the exploratory phase. The book reading sessions in all phases took place in the participants’ home and were video-recorded by the mother, who used her own videocamera device. The child was informed and agreed to be filmed too. Before the first recorded session, a filming tips handout was provided to ensure the whole interaction was captured. The filming tips were (a) use a room with good light and no noise; (b) leave the camera in a fixed place; (c) make sure to have enough memory on your card and enough battery; (d) in case of recording with a cellphone, turn off the mobile data to avoid calls or notifications while recording; and (e) verify the interaction scene is in focus. The mother shared the videos online with the researcher for later analysis and, depending on the program’s phase, for providing feedback.

#### Exploratory Phase

After the storybook selection, the mother was asked to record the storybook reading activity with her child in their home. The only instruction for this phase was: “read the storybook the way you always do with your child.”

The mother sent a total of seven videos in this phase. The first four videos were used to minimize reactivity bias, give a period for camera sensitization, and optimize recordings’ quality (e.g., camera angles, background noise, etc.). These first four videos also served to identify behaviors that might contribute new categories in the observation instrument. The last three sessions were taken for analysis.

#### Online Training Session

One online training session was offered and lasted about 1 h 20 min. Throughout the session, the clinician encouraged the mother to share her ideas, doubts, or questions. This session consisted into four stages presented below.

##### Conversation and commitment

The session started with a conversation about the interaction and emotional communication obtained through the EDEC interview and exploratory phase observations. In this stage, the researcher promoted a discussion about the importance of emotional development and its relationship with language and communication, as well as the importance of fostering emotional learning by creating environments to talk about emotions, using the storybook reading activity as an opportunity to pursue this matter. The clinician also shared the purpose of the training session, which is to receive suggestions and strategies to implement in the storybook reading activity with her son to foster conversations about emotions that can continue supporting her child’s emotional learning and development. The stage finished with asking the mother if she would like to commit to the training.

##### STEPS description

This stage aims to explain the Strategies for Talking about Emotions as Partners (STEPS) and their communicative components, including the design and use of AAC systems.

Graphic materials supported the training (see section “Materials and Methods”) to explain the steps for fostering conversations about emotions (name-cause-response): (1) Discuss the name of the emotion, (2) Discuss the possible causes for that emotion, and (3) Discuss possible responses to the emotion. The communication strategies suggested that in each step (name-cause-response), the parent: (a) Asks an open-ended question (e.g., How does Louis feel?), (b) Waits for child’s response (at least 5 seconds), and (c) Responds and provides feedback using the child’s communication system (e.g., “you’re right, Louis needs to ask for help,” selecting at the same time the words in his AAC device).

The researcher gave other types of suggestions to promote and model communication with the child. For instance, if the child does not respond after an open-ended question, provide a double-choice question by pointing to the word-choices in the child’s communication system (e.g., “Do you think Louis is angry because his friends *went away* or because he *doesn’t like Kelly*?”). If the child still does not answer, give the correct answer while modeling the communication using the child’s device (e.g., “Louis is *angry* because he *doesn’t like Kelly*”). The researcher also encourages the mother to have the child’s communication system available at all times during the session and make comments using the AAC device to model and encourage communication without requiring it.

Subsequently, an example of how to design an emotional communication board that includes the steps (label-cause-response) was presented (see section “Materials and Methods”). It was explained that the vocabulary added in the communication boards must coincide with the vocabulary used in the family and child’s context. Therefore, it is emphasized that it is of the utmost importance that the mother gets involved in the AAC board design, that is, to scan in the storybook selected, choose the situations to talk about, and write down the vocabulary she would like to discuss with her child (using a template to write it down according to the STEPS). The mother had the freedom to choose whatever emotion she would like, and in which she felt comfortable, to discuss with her son.

##### Strategy demonstration

After the STEPS description, short video demonstrations (see section “Materials and Methods”) were presented with the purpose of modeling and illustrating the strategies presented. Discussions on the strategies were encouraged, and the researcher gave in-depth explanations about the interaction and strategies performance.

##### Verbal practice and feedback

The mother was asked to describe the three steps suggested for discussing emotions she just learned, including the communication strategies to encourage emotional conversations in children with CCN (ask, wait, respond, comment, model AAC). This stage aimed to affirm and ensure the mother’s learning in the training session and give feedback.

##### Commitment to employing the strategy

At the end of the training session, the mother was asked if she would like to continue with the program and try the strategies. She responded, “yes, absolutely, it’s really fascinating and sounds so nice.” Nevertheless, she expressed possible difficulties in having the time to make the activity and record it due to different family situations. The researcher commented that the program would adapt to their needs and family time (one of the benefits of using observational designs is its applicability in natural settings and everyday life). The mother agreed to fill in the communication board design page and sent it to the researcher. Moreover, once the pages were created, she could start implementing the strategies suggested.

#### Strategy Implementation Phase

Once the AAC pages were designed, and the mother was satisfied with them, the mother video-recorded four sessions of the storybook reading activity with her child while implementing the training session’s strategies suggested. The researcher watched the recordings and gave feedback and suggestions. In this phase, the mother was also encouraged to ask questions and express her ideas about the mother–child interaction; the clinician offered a space for listening and addressing her needs, concerns, and thoughts. For example, in the beginning, she commented that it was awkward to discuss while reading “because that breaks the rhythm of the book.”

#### Iteration Phase

Three different storybooks were used in this phase. Therefore, new vocabulary, if needed, was added to the AAC pages. Three sessions of the storybook reading activity were recorded in this phase, and the mother was encouraged to continue fostering opportunities to talk and learn about emotions. The researcher gave less support; nevertheless, the mother was still encouraged to express her ideas, doubts, or questions about the interaction with her son. For example, she asked how she can encourage more discussions about her child’s emotions during the storybook.

#### Maintenance Phase

The mother asked the child which storybook he would like to read, with the possibility of choosing all the storybooks used in the program. Three storybook-reading sessions were recorded. In the maintenance phase, the researcher did not give feedback about the participants’ performance. The objective in this phase was to identify communicative changes generated by the program.

### Data Quality Control

Before data analysis was carried out, a data quality control was performed through intra-observer agreement using GSEQ 5.1 software (Bakeman and Quera, 2011). The first author recoded fifteen percent of the sessions, with at least 3 weeks of difference between the first and second codification. Cohen’s Kappa (Cohen, 1960) resulted in a satisfactory agreement average of 0.87. The sessions used for data quality control were selected randomly and using different extracts from different sessions from each of the phases in the program.

### Data Analysis

A total of 13 storybook reading sessions held over a period of 11 months were analyzed. The average observation sessions lasted 18 min, 7 s. All videos were imported and coded through Lince software (Gabin et al., 2012). The first author observed and coded each of the behaviors included in the observation instrument. The coded data considered the frequency, order, and time of each behavior observed.

Two data analysis techniques were used: (1) lag-sequential analysis and (2) polar coordinate analysis. These techniques have proven efficacy in different research areas, including individuals with special needs, such as clinical psychology (Arias-Pujol and Anguera, 2020), education (Escolano-Pérez et al., 2019), communication (Rodríguez-Medina et al., 2018), and AAC (Todman et al., 1994; File and Todman, 2002). Data were also analyzed descriptively (frequencies of communicative turns and emotional content in each phase).

#### Lag Sequential Analysis

This technique is used to identify how one or more behaviors work and presents, if any, a sequence of statistically significant actions (not due to chance) connected to specific given behaviors (i.e., the behavioral triggers that may initiate or promote a behavior pattern along time; Bakeman and Quera, 2011; Anguera et al., 2021). In other words, this analysis provides a measure of how likely is that one behavior (i.e., the “given” behavior) is followed by another [i.e., the “target” behavior(s)], either immediately (i.e., lag 1) or after two (i.e., lag 2) or more (i.e., lag 3, lag 4, etc.) successive behavioral events.

The analysis, adequate for the identification of patterns of social interactions, consists in proposing the given behavior(s), the conditioned or target behavior(s) (i.e., the actions that could be significantly associated with the given behavior), and the lag (i.e., the distances or place of order within the conditioned behavior in relationship with the presence of the given behavior). Once these criteria are defined and based on the given behavior, the matched frequencies are calculated, which is a parameter that is comprised of the number of times that a certain conditioned behavior appears before (if the lag is negative), after (if the lag is positive) or concurrently (if lag = 0) with the specific given behavior. From the matched frequencies, the expected and conditional probabilities are calculated for each lag, and adjusted residuals are obtained (Allison and Liker, 1982), revealing the likelihood of occurrence/co-occurrence of each conditioned behavior in association with the given behavior. *Z* scores are statistically significant (*p* < 0.05) for values >1.96 (i.e., the association between behaviors is activated) or <–1.96 (i.e., the relationship is inhibited). To decide when the behavioral pattern ends conventionally, the following interpretative guidelines were considered (Anguera et al., 2021): (a) when there is an absence of statistically significant behaviors in the lags; (b) when there are two successive empty lags; or (c) when in two consecutive lags, various statistically significant behaviors appeared, if so, the first of these lags is defined as the MAX LAG. Considering these guidelines are only recommendations (not compulsory criteria), when various statistically significant behaviors appear, but the significant lags after the MAX LAG were considered illustrative in understanding the mother–child communicative sequences, it was decided to incorporate the subsequent lags’ significant behaviors.

The mother’s communicative behaviors concerned with implementing the strategies suggested in the training session were selected as the given behaviors. Hence, the mother’s behaviors related to asking open-ended questions, waiting, giving the answer, providing feedback, and making comments were considered to be of special interest. Both responses from the child and mother were chosen as the conditional behaviors to identify significant interactive patterns during the conversation about emotions. A particular interest in the analysis was the child’s behaviors in discussing emotions: answering, making comments, and asking questions. The analysis only deemed the units from the observational instrument with a frequency > 4 at least in one of the phases. Values lower than 5 are considered not significant in observational methodology (Sackett, 1980).

The search for associations between the given and the conditional behaviors was made prospectively (lag 1 to lag 5) and retrospectively (lag –1 to lag –5). Concerning the retrospective analysis, only the given behaviors expected, in theory, to be the next part of a conversational sequence already begun (e.g., providing feedback, giving the answer) are presented in the results section. The child and mother’s utterances and their simultaneity with the type of emotional content discussed and method of expression were also analyzed (in lag 0). The lag-sequential analysis was applied to each of the program phases to identify communicative patterns among them.

#### Polar Coordinate Analysis

Polar coordinate analysis (Sackett, 1980) is performed to identify a representative map that explains the type of relationship between a focal behavior (i.e., the behavior of interest) and the selected conditioned behaviors (i.e., actions that could be associated with the focal behavior). This technique employs the adjusted residual values obtained in the lag sequential analysis. It integrates both prospective (e.g., lag 0 to +5) and retrospective (e.g., lag 0 to –5) perspectives, which are used to calculate the Zsum scores (prospective and retrospective), as well as the vectors (length and angle) for each conditioned behavior. For this analysis, the genuine retrospectivity proposed by Anguera (1997) was used. Each conditioned behavior can be represented graphically; depending on the quadrant in which the vector is located, the relationship between the focal and conditioned behavior is interpreted (activation vs. inhibition):

• Quadrant I: Both behaviors (focal and conditioned) are mutually activated (prospective and retrospectively).
• Quadrant II: The focal behavior inhibits the conditioned behavior, whereas the conditioned behavior activates the focal one (prospective inhibition/retrospective activation).
• Quadrant III: The focal and conditioned behaviors are mutually inhibited (prospective and retrospective inhibition).
• Quadrant IV: The focal behavior activates the conditioned behavior, whereas the conditioned inhibits the focal one (prospective activation/retrospective inhibition).

The behaviors that were suggested to be implemented in the training session to the mother, and that showed a frequency > 4 at least in one of the phases, were identified as the focal behavior: ask, make comments, respond, model AAC communication; while the child’s communicative behaviors: answers, makes comments, asks questions, expressing with conventional gestures or AAC were selected as the conditioned ones.

### Social Validation

Mother and child satisfaction surveys were completed at the end of the maintenance phase to evaluate the program’s social validity. The questionnaire included multiple-choice and open-ended questions about their ideas and opinions about the program’s process and participation. The child answered with his AAC device.

## Results

### Development of Emotion-Related Conversations

**FIGURE 3:**
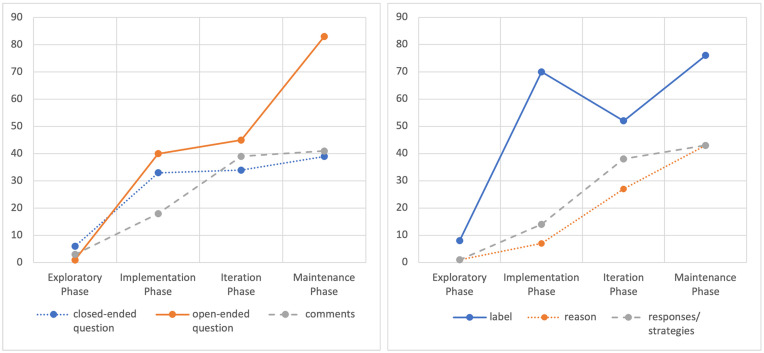
Development of the mother’s conversational utterances about emotions [closed-ended questions, open-ended question, comments] and type of emotional content discussed [label, reason, and responses to emotion].

**FIGURE 4:**
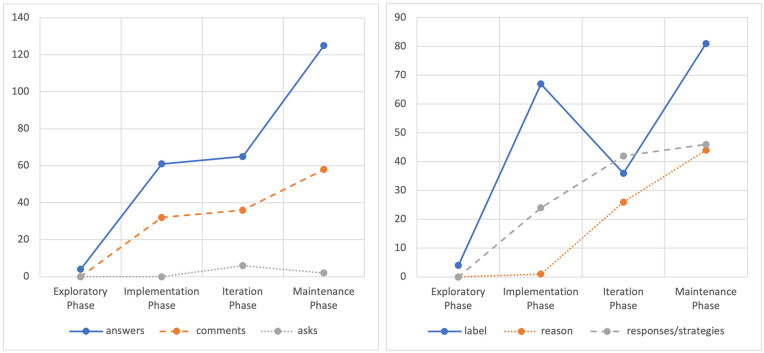
Development of the child’s conversational utterances about emotions [answers, asks questions, comments] and type of emotional content discussed [label, reason, and responses to emotion].

Figures 3 and 4 illustrate the interactive communication progress (frequencies) per phase, between the mother and her child, regarding their participation and emotional content discussed (emotional label, causes, and responses to emotions) in the storybook-reading activity. It can be noticed that in the exploratory phase, there is little stimulation in both participants about having an emotional talk. The sessions that followed the training session showed maintained progress by both the child and mother, concerning their active participation in conversations about emotions.

**FIGURE 5:**
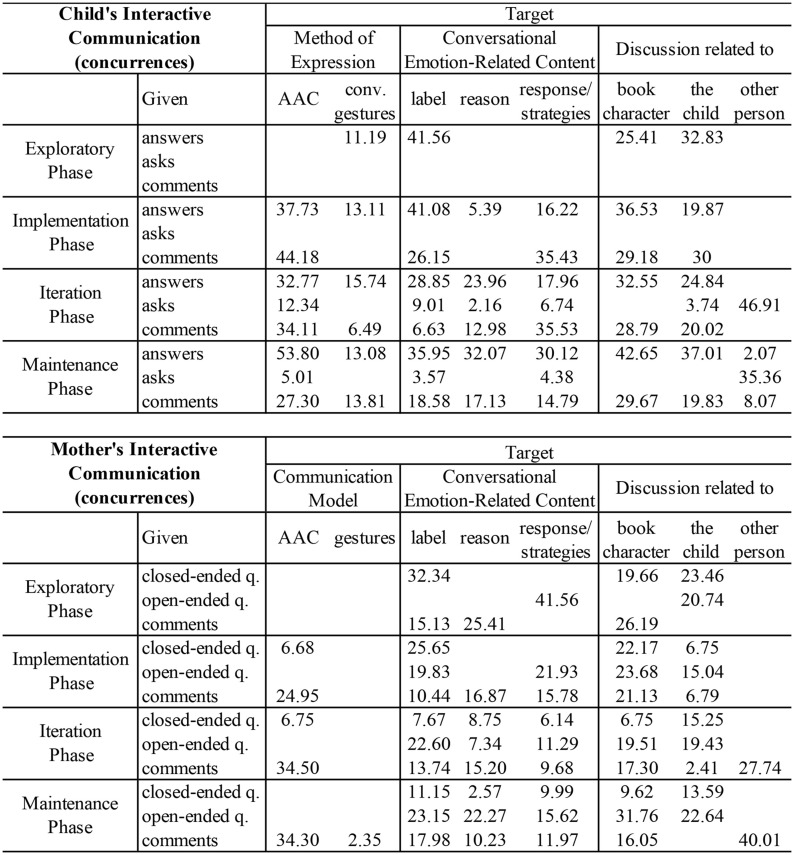
Adjusted residuals showing significant concurrences (lag 0) between emotional conversations utterances from the mother and child, and their modes of expression, and type of emotional content discuss (content and related to).

To examine the participants’ utterances in conversations about emotions, the type of emotional content (i.e., emotion label, cause, response), ways of expression (i.e., method of expression in the child and communication model in the adult), and to whom the emotional discussion was addressed (i.e., the child, storybook characters, or other people), a lag sequential analysis was performed in lag 0. Lag 0 indicates a simultaneous appearance of the selected behaviors. The results revealed highly significant concurrences (>1.96, *p* < 0.01) between these dimensions (Figure 5), demonstrating that discussions richer in emotion-related content and about different referents appeared as conversations developed over time. Additionally, it is noticeable that, after the training session, the mother showed AAC models while commenting about emotions, and the child participated in emotional discussions using his AAC and conventional gestures.

The exploratory phase was characterized by the child’s gestural responses to the mother’s questions about the emotional label from the storybook’s characters and the child. After the training session, the mother and his child showed engagement in discussions related to more than just labeling emotions (they discuss the reason and responses to emotions) about the storybook and the child. In the iteration and maintenance phases, the child’s interest in asking questions about emotions referring to himself (e.g., what can I do?) and his mother (e.g., how do you feel?) emerged.

### Behavioral Sequential Patterns of Mother–Child Interaction When Fostering Conversations About Emotions

**FIGURE 6:**
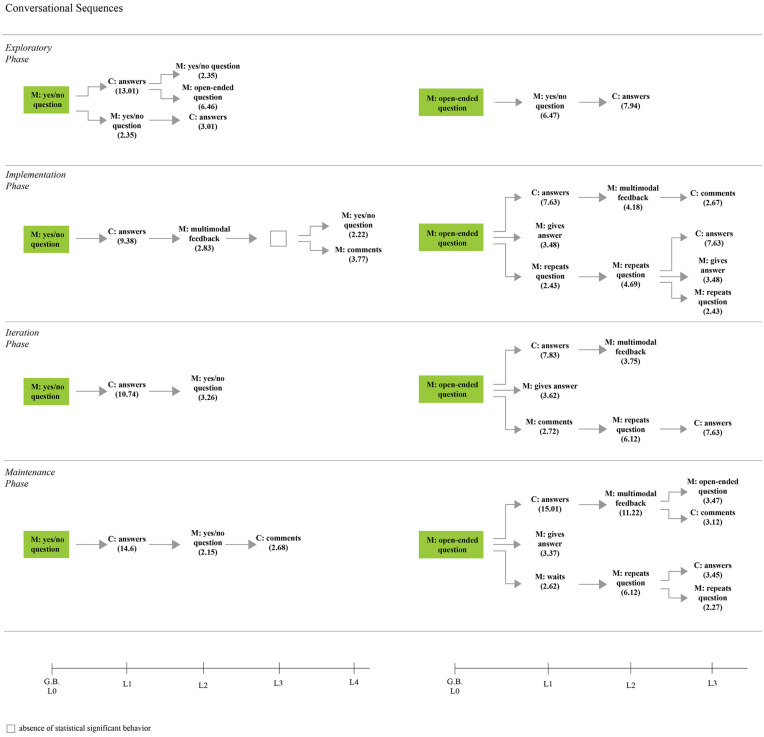
Sequential communicative patterns obtain in each phase during the storybook reading with the mother’s closed-ended and open-ended emotion-related questions as given behavior [M, Mother; C, Child].

**FIGURE 7:**
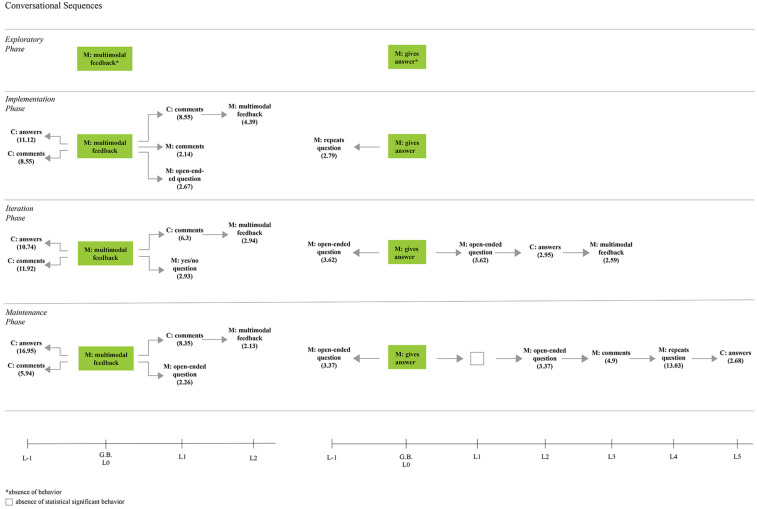
Sequential communicative patterns obtain in each phase during the storybook reading with the mother’s providing feedback and answers as given behavior [M, Mother; C, Child].

**FIGURE 8:**
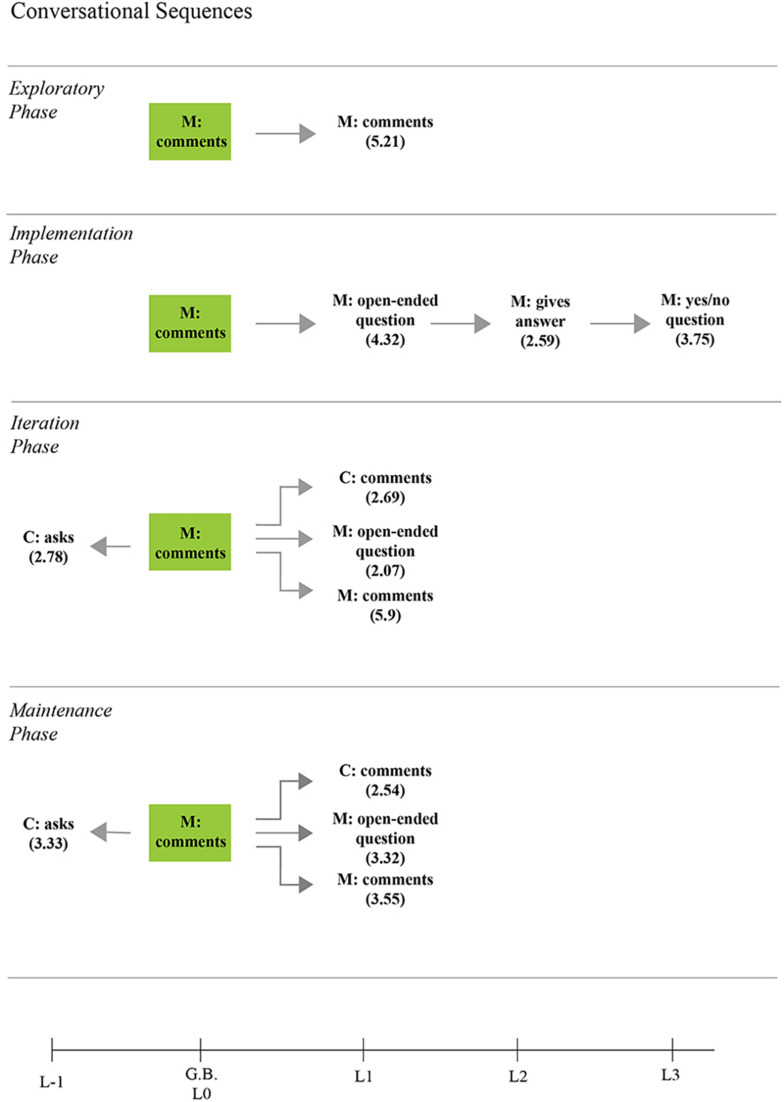
Sequential communicative patterns between mother–child during storybook reading with the mother’s emotion-related comments as given behavior [M, Mother; C, Child].

Figures 6–8 present the statistically significant sequential communicative patterns related to the dyad interaction during the storybook-reading activity in each phase. Only the patterns that showed activation (i.e., *Z* > 1.96, *p* < 0.05) between the given (the mother’s behaviors that encourage emotional conversations) and conditioned behaviors (the child’s behaviors in emotional conversations, as well as the mother’s behaviors that encourage emotion-related conversations) are presented.

#### Encouraging Child’s Participation Openly

Even though the closed-ended questions were not part of the training sessions’ suggestions, it was considered important to present them in the results section as closed-ended questions are part of the communication flow during any conversation. Yes-no questions about emotion-related events were followed by a stable behavior pattern of the child’s response, succeeded by another mother’s query in all of the phases (Figure 6). One exception to this appeared in the implementation phase, which was followed by the mother’s feedback and then another closed-ended question or comment about emotions. In the exploratory phase, the mother showed a pattern of asking more than one closed-ended question at a time, followed by the child’s response. A significant change in the pattern was shown in the maintenance phase where the child, after a second closed-ended question, tended to respond with also a spontaneous comment:

[Talking about character’s feelings]
M: Mmm, I wonder why, do you know why?
C: Stick tongue [gesture for no]
M: Or do you think he is gonna be angry?
C: Looks up [gesture for yes], confused [selected via AAC]
M: Yes, and he is confused.

A considerable difference before and after the training session can be seen when the mother asked emotion-related open-ended questions (Figure 6). In the exploratory phase, even though the mother asked open-ended questions, it was immediately followed by a closed-ended question (e.g., What do you do when you are scared? Can I see a face that you think is scared?). Nevertheless, in the phases following the training session, significant combinations of conversational turns about emotions were observed when the mother asked an open-ended question: (1) the child engaged actively in the conversation by responding (answers), and this behavior was followed by the mother’s feedback; (2) The mother, after questioning, answers immediately, and (3) Repeats the question, which in turn could finish the sequence with the child’s response. The following clinical vignette, taken from the implementation phase, demonstrates the first sequence explained above:

M: So, when somebody is so sad, what they can do?
C: Need a hug [AAC]
M: Oh, need a hug, yes, he was so sad that he needs a hug, ok.

An example of the behavioral sequence of open-ended question – mother’s comment – repeat question – child answers observed in the iteration phase would be:

M: And when you feel stressed, what should you do?
M: Cause when you feel stressed your body tenses up
M: So, if you feel stressed, what should you do?
C: [child smiles] Cheer up mate! play [AAC]
M: Oh, I know, you want someone that says cheer up! And you wanna play.

#### Answering and Giving Feedback

Answering and giving feedback are behaviors expected to be contingent on previous actions; thus, significant behavioral patterns observations from the retrospective (e.g., lag –1 to –5) and prospective (lag + 1 to 5) analysis were included in this section (Figure 7).

Feedback providing utterances significantly changed and were maintained after the training session. In the exploratory phase, this behavior related to emotional content was not observed, whereas in subsequent phases it was preceded by the child’s emotional comments and responses, and feedback, in turn, activated another child’s emotional comments:

[Discussing character’s emotions]
C: Overwhelmed
M: Overwhelmed, yes, it’s too much [for the character]
C: Surprise
M: Yes, he is probably surprised, because he lost his M.

In other cases, offering feedback activated another mother’s query or emotional comment (the mother’s comment was only significant in the implementation phase).

The mother’s expression of answering her questions was not directly associated with the child’s response, neither retrospectively nor prospectively. In phases 3 and 4, after the mother answered, she made another open-ended question encouraging the child’s conversation:

[discussing storybook]
M: They were what?
M: Maybe there is not an emotion, but they were safe
C: Looks up [gesture for yes]
M: So, they are now rescued. How do you think they felt?
C: Glad, happy [AAC]
M: Absolutely, yes, that’s great. So, they were so happy now because they were safe.

#### Commenting to Encourage Child’s Participation

The mother’s emotional comments were observed in all phases (Figure 8), with a simultaneous mother’s use of AAC in the phases after the training session (remember lag 0, Figure 3). During the phases 1 and 2, the mother’s personal comments about emotions were not prospectively associated with a significant child’s communicative behavior, whereas, by phases 3 and 4, the mother’s emotional opinions were followed by the child’s comments about emotion-related events:

[Talking about what the child can do when he feels sad because his body tenses up]
M: Maybe you can “wait” [AAC] a little bit and “breathe” [AAC]
C: Try again [AAC]
M: Yes, you can also try again.

In phases 3 and 4, the mother’s emotional comments were significantly preceded by child’s questions:

[Talking about what can the child do if he were the book’s character]
M: What can you do?
C: I need a break [AAC]
M: Yes, sure, you will need a break from all these crayons
C: What would you do? [AAC]
M: If I were him, I would say, “good job” guys, “thank you” for your service, and then I will say I will “think of a strategy” [AAC].

### Relationships Between Mother–Child Interactive Communication About Emotions

**FIGURE 9:**
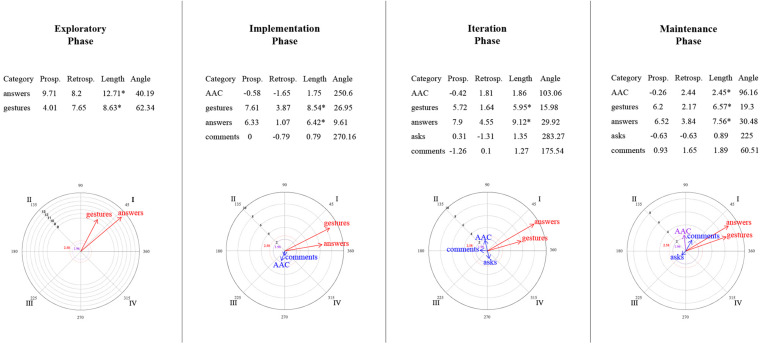
Vectors corresponding to the mother’s emotion-related closed-ended question as focal behavior, and the child’s emotion-related communicative turns [answers, comments, asks] and methods of expression [AAC, conventional gestures] as conditional behaviors.

**FIGURE 10:**
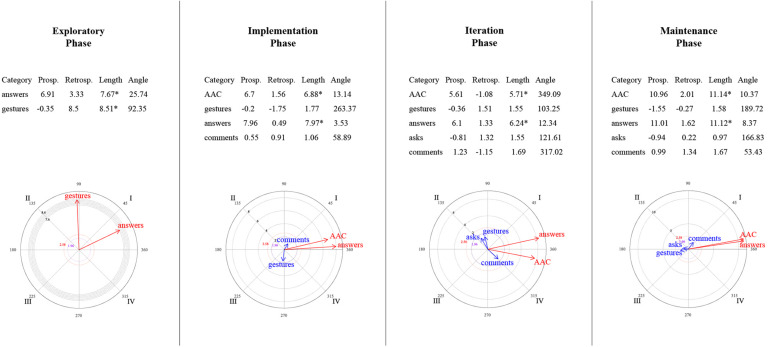
Vectors corresponding to the mother’s emotion-related open-ended question as focal behavior, and the child’s emotion-related communicative turns [answers, comments, asks] and methods of expression [AAC, conventional gestures] as conditional behaviors.

**FIGURE 11:**
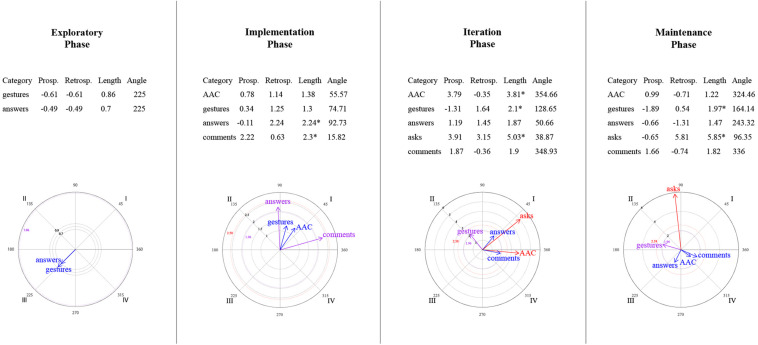
Vectors corresponding to the mother’s emotion-related comments as focal behavior, and the child’s emotion-related communicative turns [answers, comments, asks questions] and methods of expression [AAC, conventional gestures] as conditional behaviors.

**FIGURE 12:**
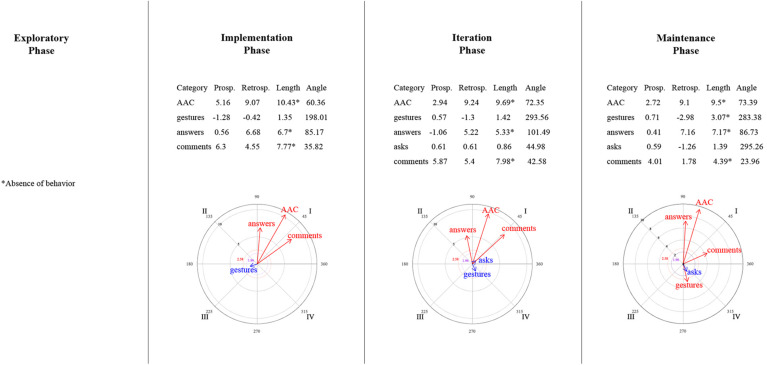
Vectors corresponding to the mother’s providing multimodal feedback as focal behavior, and the child’s emotion-related communicative turns [answers, comments, asks] and methods of expression [AAC, conventional gestures] as conditional behaviors.

**FIGURE 13:**
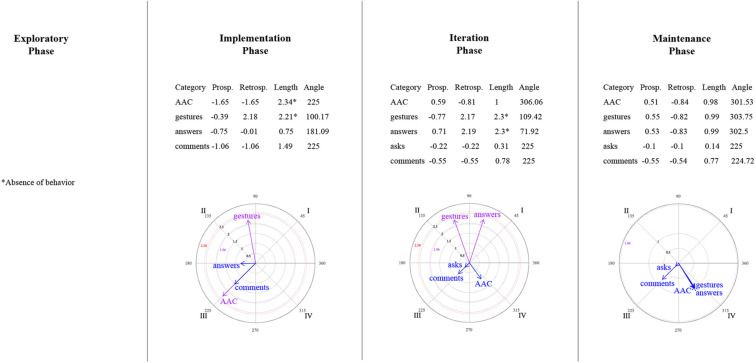
Vectors corresponding to the mother’s giving of the answer to her own questions as focal behavior, and the child’s emotion-related communicative turns [answers, comments, asks] and methods of expression [AAC, conventional gestures] as conditional behaviors.

**FIGURE 14:**
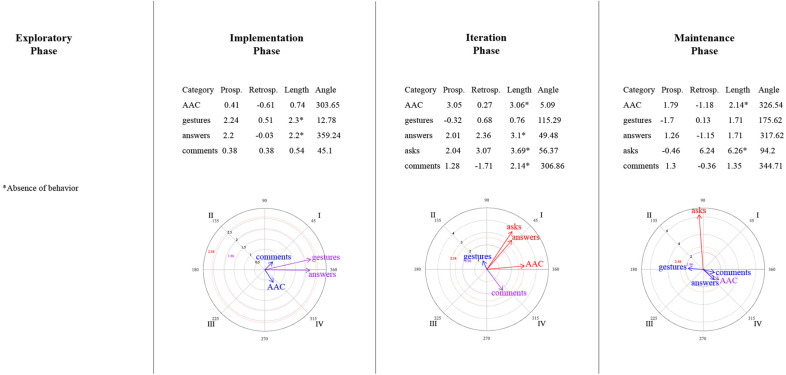
Vectors corresponding to the mother’s AAC modeling as focal behavior, and the child’s emotion-related communicative turns [answers, comments, asks] and methods of expression [AAC, conventional gestures] as conditional behaviors.

Figures 9–14 show the vectorial graphs from the polar coordinate analysis in each phase. Those graphs present the relationships between mother-child interactive emotion-related conversations. Behaviors taken as focal were mother’s closed-ended question, open-ended question, multimodal feedback, give answer, emotional comment, and AAC model. The child’s answers, comments, questions, and modes of expression were selected as the conditioned behaviors. In this section, only the vectors with significant results will be discussed (i.e., with a length > 1.96, *p* < 0.05, are represented in purple; vectors with a length > 2.58, *p* < 0.01 are represented in red). Vectors in blue are not significant.

#### Mother’s Questions and Child’s Engagement in Conversations

Similar to what was found in the lag-sequential analysis, Figure 9 shows the significant stable mutual activation (Quadrant I) in all of the program phases between the mother’s closed-ended questions and the child’s answers through gestures. The child’s AAC mode of expression in the maintenance phase was also significant and located in quadrant II, indicating that closed-ended questions inhibit the child’s use of AAC, whereas this expression activates mother’s closed-ended questions.

Concerning open-ended questions (Figure 10), a stable mutual stimulation with the child’s emotion-related answers was identified during all of the phases. Nevertheless, changes were found in terms of the child’s modes of expression. In the exploratory phase, the mother’s open-ended questions inhibit the child’s gestures, but this behavior, in turn, activates the mother’s questioning. A different pattern was observed after the training session, where the child’s AAC use mutually activate the mother’s open-ended questions in phase 2 and 4. In contrast, in phase 3, the child’s AAC use is situated in quadrant IV, indicating that the mother’s open-ended questions activate the child’s AAC expressions, but the AAC use inhibits the mother’s open-ended questioning.

#### Mother’s Emotional Comments and Child’s Engagement in Conversations

No relationship was found in the exploratory phase between the mother’s emotional comments and the child’s behaviors (Figure 11). Phase 2 presented a mutual excitatory association between the mother’s comments and the child’s emotional comments. However, in phase 3, the reciprocal activation was between the mother’s comments and the child’s questioning. By phase 4, this communicative link changed to quadrant II, implying that the child’s inquiry about emotions stimulates the mother’s comments but not vice versa. A similar relationship was observed with the child’s gestures (quadrant II) in phases 3 and 4; that is, the mother’s comments inhibit the child’s gestures, but those gestures activate the mother’s comments. Finally, the AAC child’s mode of expression was significantly associated with the mother’s comments in the iteration phase (quadrant IV), where the focal behavior stimulates the child’s AAC use but not conversely.

#### Mother’s Feedback/Answer and Child’s Engagement in Conversations

A significant relationship was found between the mother’s multimodal feedback and the child’s behaviors after the training session (Figure 12). Feedback was strongly mutually activated with the child’s emotional comments in all phases, and with the child’s responses to questions in phases 2 and 4. In phase 3, the mother’s feedback inhibited the child’s answers, but this response activated the mother’s feedback.

Concerning the mother’s answering of her questions (Figure 13), in phase 2, the child’s AAC use and the mother’s giving the answer inhibited each other (Quadrant III). At the same time, the child’s gestures activated the mother’s giving the answer (a similar association was also observed in phase 3). In addition, significant excitatory association was noted in the iteration phase between the child’s response and the mother’s answering. Phase 4 did not present a significant relationship between the focal behavior and the child’s behaviors or expression methods.

#### Mother’s AAC Modeling and Child’s Engagement in Conversations

In the exploratory phase, no AAC model was presented (Figure 14). Nevertheless, in the implementation phase, the mother’s use of AAC, while discussing emotions, involved mutual activation with the child’s gestures and prospective activation with the child’s emotional answers to the mother’s questions.

By the iteration phase, modeling AAC showed a reciprocal activation with the child’s answers, questions, and AAC use to communicate emotion-related events; and a unilateral activation with the child’s comments. However, these relationships were not sustained in the maintenance phase. It was observed that the child’s questions stimulated the mother’s AAC model, and the mother’s AAC model activated her child’s AAC use, but none of them conversely.

### Mother–Child Social Validation

A written satisfaction survey was sent to the mother and her child by the end of the maintenance phase to evaluate the training and program’s social validity.

The mother expressed feeling extremely satisfied with the support provided during the program and considered it helpful and very easy to learn emotional communication strategies. She commented that participating taught her:

how to have a deeper conversation with my son. To be mindful of the characters in the books and use them as a tool to talk to my son (…) this [the emotion communication strategy learned] enables otherwise a superficial, two-dimensional conversation to be more interesting. I got to learn more about how my son feels. (…) Got to understand more about the importance of discussing the emotions and how to deal with the emotions. (…) [the “how to respond” page] has helped my son to also think deeper. [this program] opens up many more opportunities to use AAC and talk about more abstract issues (not just factual questions).

The child indicated that he enjoys talking about emotions in the storybook reading activity “a lot” and that he learned something new about emotions: “(I learned) to ask questions like How are you? To answer like fantastic. To communicate.” He also shared that he “absolutely” likes to talk with his mom using his AAC device, and what he likes the most about the emotion-related AAC pages is “to be able to express I like, I love,” whereas what he like the least was “dizzy – too many choices.” When asked to complete the sentence “I want to say that…,” he commented, “Ready and sharing – Enjoyed.”

## Discussion

The present study highlights the importance and promising implications of providing interactive learning experiences in natural settings to encourage emotional conversations in children with CCN. Similar to the findings presented by Na and Wilkinson (2018), participating in the program resulted in improvement in the communicative exchanges between mother–child about emotion-related events.

During the exploratory phase, the child rarely had opportunities to discuss emotions, and his participation was mainly summarized in answering yes/no questions. Although the mother occasionally promoted a richer emotional discussion (asking open-ended questions), this was followed by a closed-ended question. Research has shown that asymmetries between discourse patterns between partner-individual with CCN are frequently expected (Todman et al., 1994) and that there is a tendency to engage individuals with CCN through yes/no questions in communicative exchanges as it speeds up the interaction. Nevertheless, asking closed-ended questions limits their experience and opportunities to learn, discuss and interact actively (Light et al., 1985; Beukelman and Light, 2020).

After the training session, a considerable improvement in the child and mother’s utterances and communicative patterns was observed. The mother’s prompts to encourage the child’s involvement in conversations about emotions, as well as the proper culturally sensitive AAC system design, facilitated the child’s active participation during the storybook-reading activity. The availability of emotion-related vocabulary contributed to the child’s ability to sustain and start conversations about emotions. The behavioral patterns obtained permitted analyzing the communicative change over time between mother–child emotional conversations. Maintaining the mother’s prompting to foster emotional talk helped increase the child’s conversational contributions substantially in terms of making spontaneous comments rather than just responding to questions, asking questions about emotion-related events to others, and talking about himself rather than only the storybook’s characters.

The analysis carried out allowed for the identification of what types of the mother’s behavior encourage or inhibit particular behaviors by the child. For example, it was noticeable how the conversational sequence and relationship with the child’s behavior changed during the program phases around the mother’s emotional comments. Even though the mother made emotional comments during the exploratory phase, the child did not show any conversational response. In contrast, in the following phases, when she commented about emotions, concurrently with modeling the use of AAC, the child showed interactive behaviors that were significantly connected to that mother’s prompt. These findings are consistent with the literature that highlights the importance of supporting communication partners in providing models and opportunities, deliberately, to foster interaction and development of emotional and communicative competencies in individuals with CCN (Biggs et al., 2018; O’Neill et al., 2018; Wilkinson et al., 2021).

The present case study sheds light on the promising efficacy of supporting communication partners online in creating interactive learning environments at home to encourage emotional and communication skills to discuss emotions, while respecting the family conditions and cultural background. At the beginning of the program, the mother expressed some concerns about engaging in the intervention due to specific family situations and difficulties in having the time to make the activity and record it. Despite the family time barriers to engaging in the program within a brief period [e.g., less than 3 months as in Na and Wilkinson (2018)’s multiple-baseline research design], the intervention still showed positive results. Sometimes, family effective engagement in interventions may be hindered by logistical barriers like parents’ work schedules (Brotman et al., 2011). Being flexible to the context and understanding the child with CCN, family, and socio-cultural needs and interests are essential in creating appropriate and sensitive interventions that support children’s learning (Rangel-Rodríguez et al., 2021). Systematic observation approaches provide the flexibility needed to studying natural settings and everyday life without losing rigor in the investigation (Anguera et al., 2018).

An evident drawback of the study was the limited number of participants. However, the observational methodology employed in the present study allows intensive research, being inversely related to the extensiveness required by other methodologies. Moreover, single case studies are the best path to follow when the topic to be studied is emerging (Swanborn, 2010). The analysis used illustrates a novel approach for conducting single case studies in the field of communication (including AAC), psychology, and education. Polar coordinate analysis and lag sequential analysis provide an innovative way to model the conversational pathways that change over time after an intervention in everyday contexts. These analyses offer information on the relationship and sequences between behaviors that cannot be understood through other conventional analyses, such as those that measure the frequency of appearance of a target behavior.

Further research is needed to continue validating the intervention and involving more children with CCN, from different socio-cultural backgrounds and linguistic levels. Additional work is warranted to identify the generalization of the emotion conversational abilities obtained in other settings outside the storybook-reading activity. The mother commented they had conversations about emotion-related events outside the storybook-reading sessions, where the child accessed his AAC emotional communication pages to discuss specific events. Further studies are relevant to adapt the program in other contexts, such as including both parents, siblings, or group settings (e.g., at school, group therapy); and in other activities, such as role-playing, watching movies or series, playing games, etc.

To sum up, significant communicative changes between the mother and child occurred in the interaction when the mother encouraged opportunities to discuss emotions in a storybook-reading activity. The present findings support the promising outlook of providing interactive home learning environments to foster emotional talk in children with CCN who may benefit from AAC.

## Data Availability Statement

The datasets generated for this study are available on request to the corresponding author.

## Ethics Statement

The study was reviewed and approved by the Ethics Committee of the Universitat Autónoma de Barcelona. Written informed consent to participate in this study was provided by the participants’ legal guardian/next of kin.

## Author Contributions

GR-R documented, designed, developed the project, carried out the study, and wrote the manuscript. MB and SB made direct contributions to the work, revised the manuscript, and approved it for publication. All the authors contributed to the article and approved the submitted version.

## Conflict of Interest

The authors declare that the research was conducted in the absence of any commercial or financial relationships that could be construed as a potential conflict of interest.

## Supplementary Material

Supplementary Material 1 STEPS Instruction Page (adapted).

Supplementary Material 2 Example of AAC emotion-related boards.

Supplementary Material 3 Observation Instrument for interactions between communication partners and children with CCN during a storybook-reading activity (full version).
