# Supplementary material for: Encouraging Emotional Conversations in Children With Complex Communication Needs: An Observational Case Study
Source: Front Psychol. 2021 Jul 6;12:674755. doi: 10.3389/fpsyg.2021.674755 (PMC8290146; doi:10.3389/fpsyg.2021.674755)
Supplement: Supplementary Material 1 — STEPS Instruction Page (adapted). [file Data_Sheet_1.PDF]

STEPS Training

Strategies to Talk about Emotions as Partners

Instruction Page

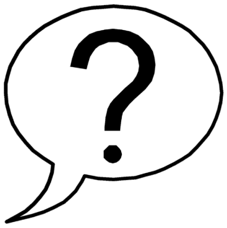

Ask

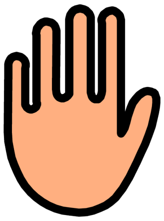

Wait

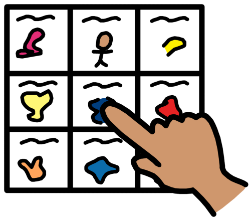

Respond

	Ask	Wait	Respond	
	Allow the board to be within reach of your child, but don't give him clues (e.g. pointing directly to the board).<br><br>You can repeat/rephrase your question 1-2 times to help your child understand it.<br><br>If necessary, you can provide double-choice questions (e.g. is the lion sad or angry?) by pointing to the images on the board.	Pause for at least 5 seconds or until your child begins to respond.<br><br>Look directly at your child to convey an expectation that it is his turn in the conversation.<br><br>Allow the emotional communication board to be within reach of your child, but do not provide clues (e.g. pointing directly to the board).	If your child answers properly, provide active listening (repeat what your child said) while pointing to the answer on the communication board.	If your child answers something different or does not respond after 10 seconds, give the correct answer while pointing to the corresponding symbol on the communication board.
**1. Talk about the NAME of emotion**	**How** does the lion feel?	… pause…	You're right, the lion is sad<br><br>**pointing the word(s) on the board**	The lion is sad
**2. Talk about the CAUSE of emotion**	**Why** is the lion sad?	… pause…	You're right, the lion is sad because he has no friends<br><br>**pointing the word(s) on the board**	The lion is sad because he has no friends
**3. Talk about the RESPONSE to that emotion**	**What** can the lion do?"	… pause…	You're right, the lion can use his words and ask them to play together<br><br>**pointing the word(s) on the board**	The lion can use his words and ask them to play together

*Remember that you can comment using the board to model and encourage communication without requiring it.

The Picture Communication Symbols ® and Boardmaker by Tobii Dynavox® All rights reserved. Used with permission.

Adaptation from Na & Wilkinson (2018)
