# Supplementary material for: Encouraging Emotional Conversations in Children With Complex Communication Needs: An Observational Case Study
Source: Front Psychol. 2021 Jul 6;12:674755. doi: 10.3389/fpsyg.2021.674755 (PMC8290146; doi:10.3389/fpsyg.2021.674755)
Supplement: Supplementary Material 2 — Example of AAC emotion-related boards. [file Data_Sheet_2.PDF]

Example of AAC emotion-related boards

Main List: Personal (1)

PCS® and Boardmaker by Tobii Dynavox®
All rights reserved. Used with permission.

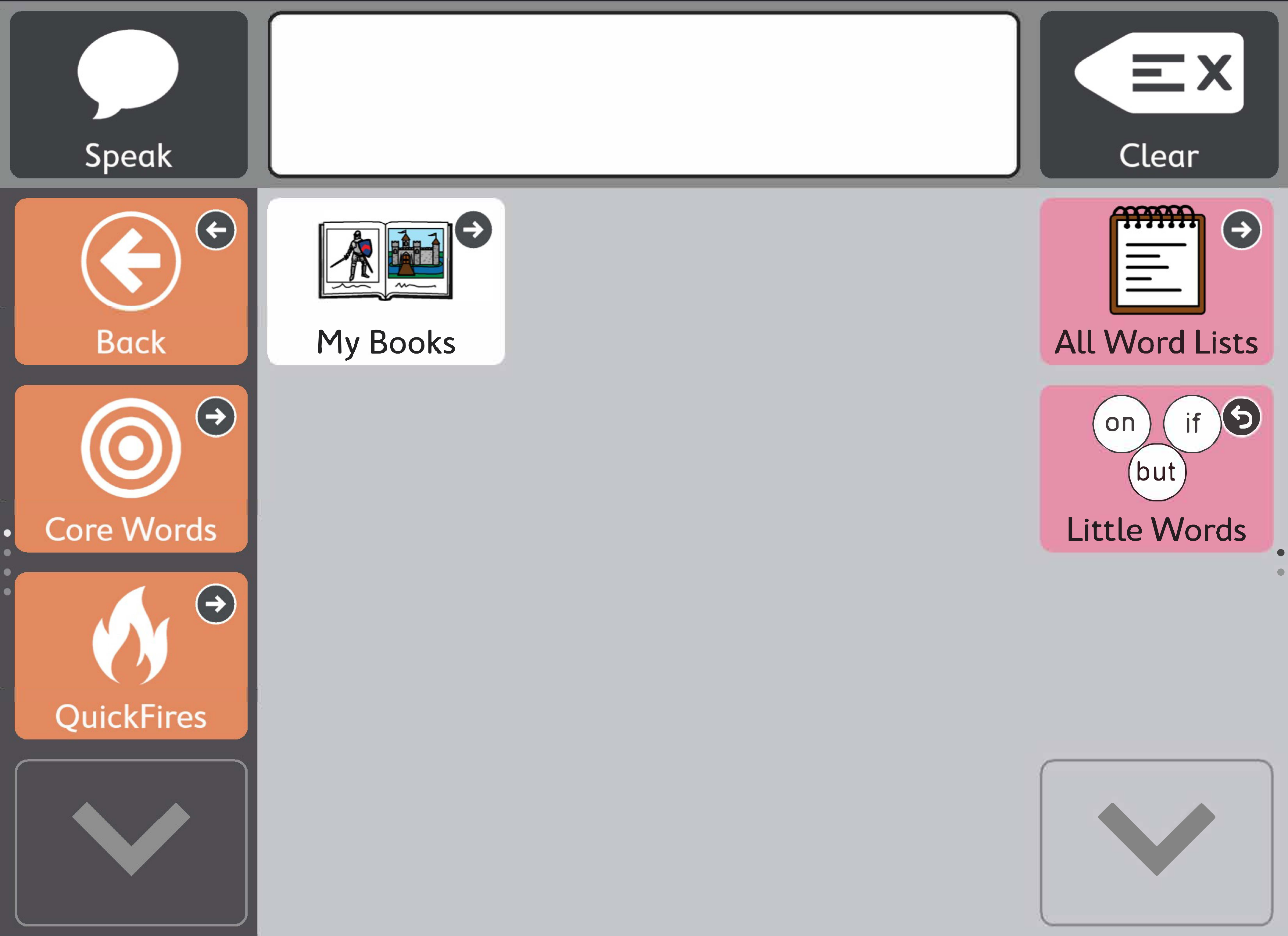

Example of AAC emotion-related boards

My books

PCS® and Boardmaker by Tobii Dynavox®
All rights reserved. Used with permission.

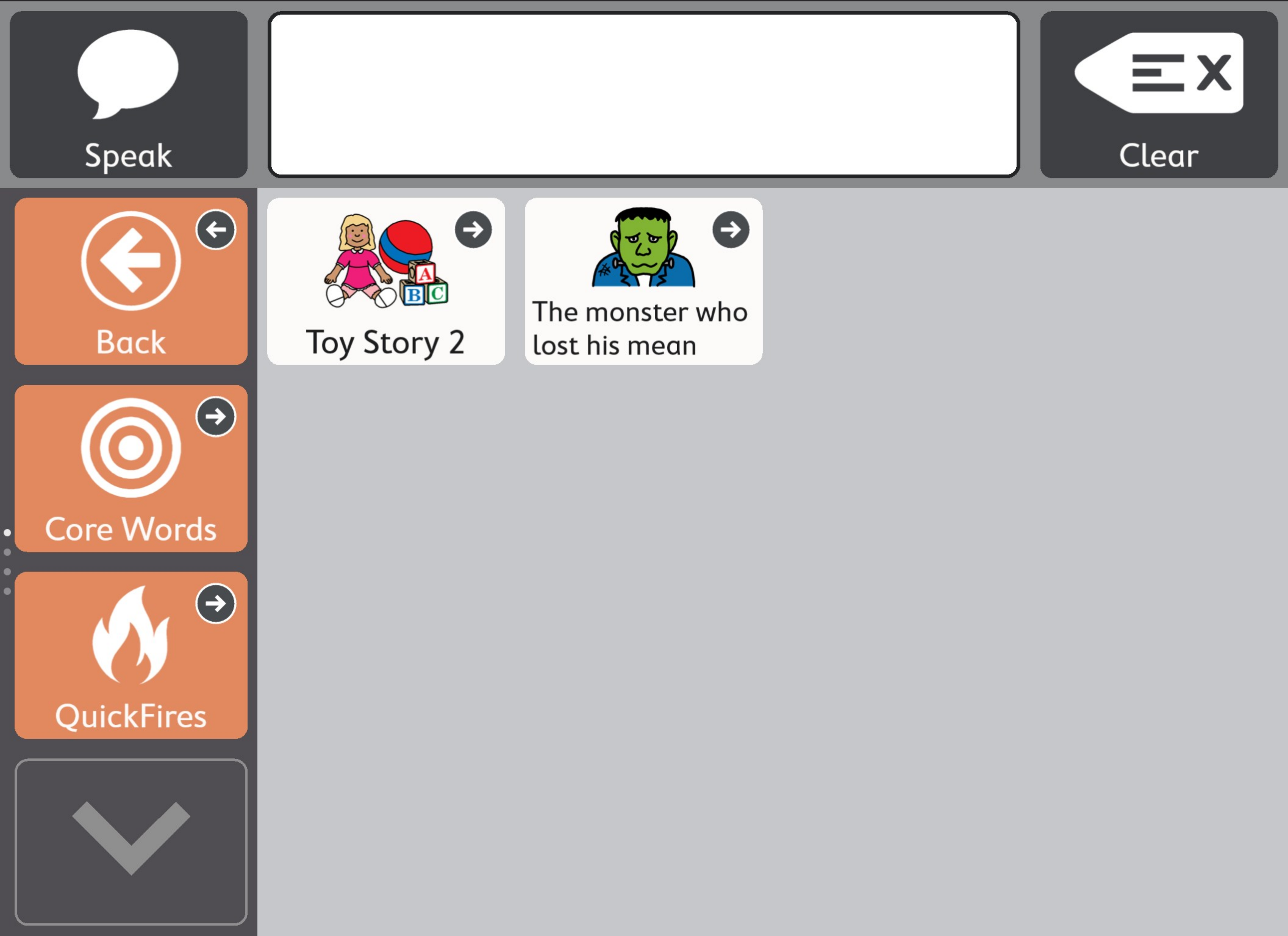

Example of AAC emotion-related boards

Toy Story

PCS® and Boardmaker by Tobii Dynavox®
All rights reserved. Used with permission.

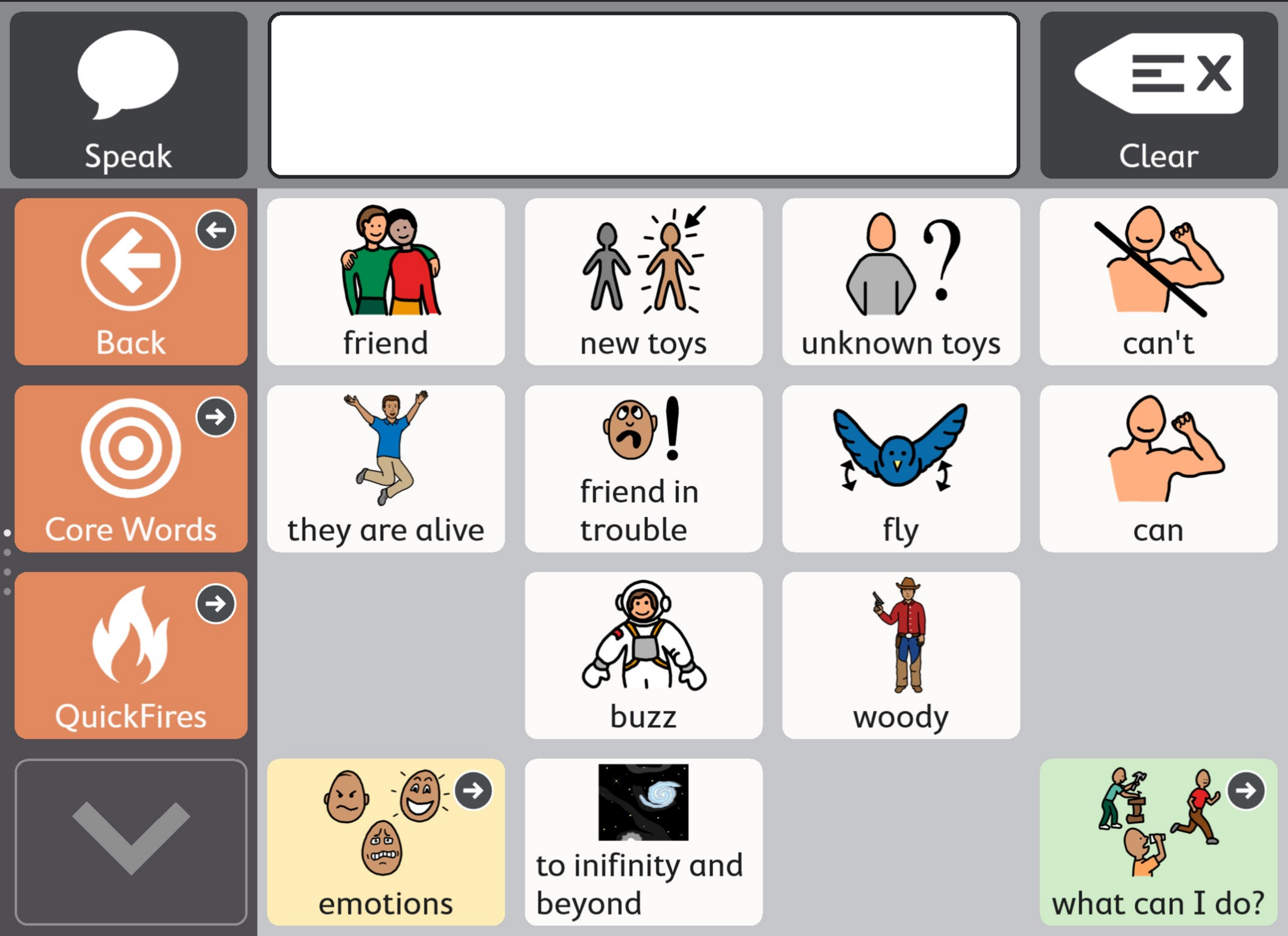

Example of AAC emotion-related boards

Main List: Feelings (1 / 2)

PCS® and Boardmaker by Tobii Dynavox®
All rights reserved. Used with permission.

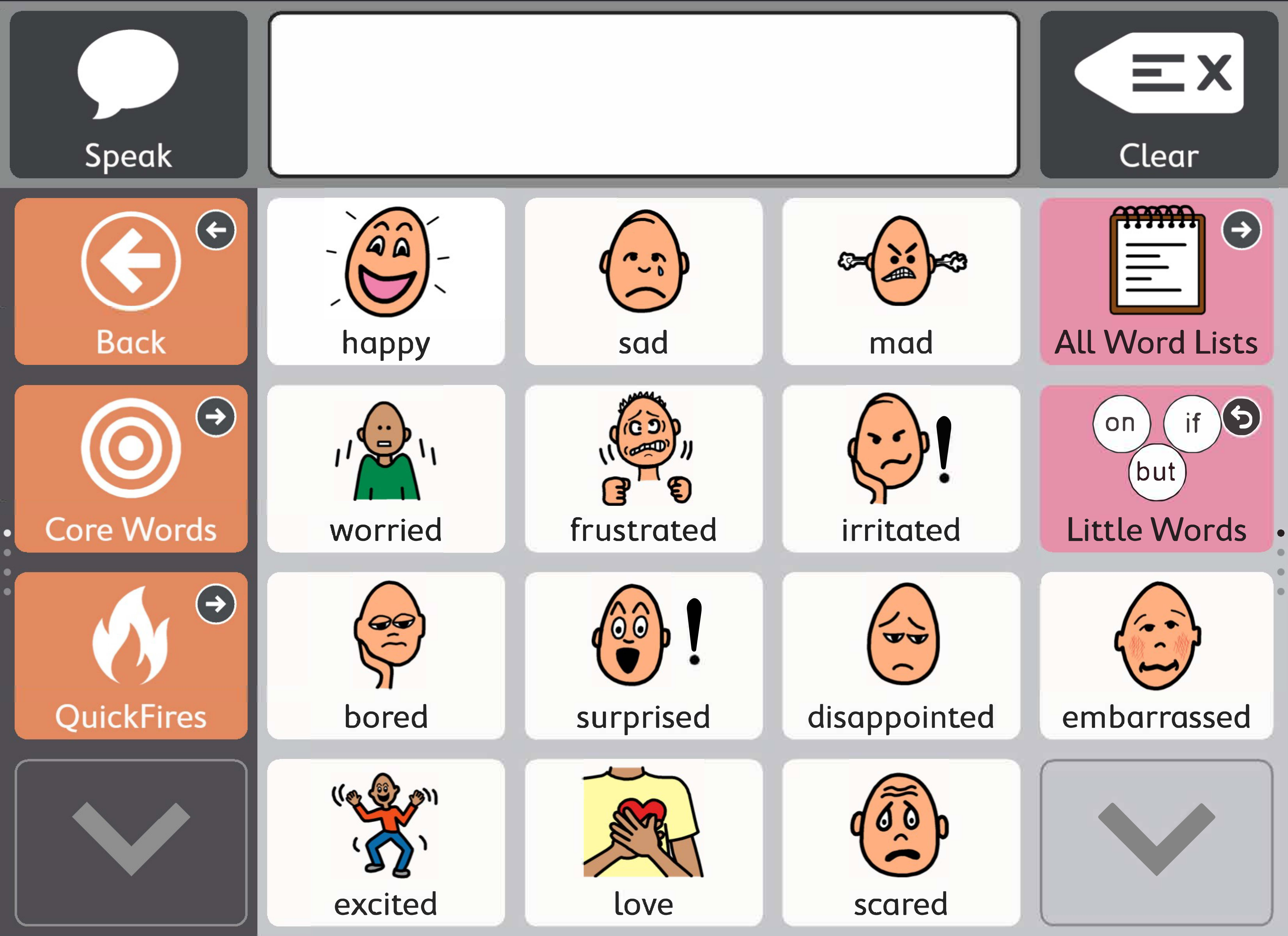

Example of AAC emotion-related boards

Responses (1 / 2)

PCS® and Boardmaker by Tobii Dynavox®
All rights reserved. Used with permission.

Speak				Clear
Back	cry	need a hug	go somewhere else	I don't like that
Core Words	seek	hide	cheer up mate!	need help
QuickFires	do something fun	play	enjoy	I want to talk to someone
	thank you!	I love you!	laugh	
