# Supplementary material for: Encouraging Emotional Conversations in Children With Complex Communication Needs: An Observational Case Study
Source: Front Psychol. 2021 Jul 6;12:674755. doi: 10.3389/fpsyg.2021.674755 (PMC8290146; doi:10.3389/fpsyg.2021.674755)
Supplement: Supplementary Material 3 — Observation Instrument for interactions between communication partners and children with CCN during a storybook-reading activity (full version). [file Data_Sheet_3.PDF]

Observation Instrument for Interactions between Communication Partners and Children with CCN during a Storybook-Reading Activity (full-version)

Rangel-Rodríguez G.A., Badía, M., & Blanch, S.

Macro-dimension	Dimension	Subdimension/Units (codes)/Examples
Adult's Interactive Communication	Behaviors that encourage conversations	Encourages participation openly*<br>closed-ended question (acQ): Do you think he is sad?<br>open-ended question (aoQ): Why is she angry?<br>two choices (aTC): Do you think the wizard is scared or surprised?<br>sentence completion (aSC): The mouse feels excited because…<br>multiple choices (aMC): does the horse need to take a break, ask for help, or explain how he/she feels?<br>repeat question (aRQ)<br>Turn-taking signal*<br>indirect turn (IT): waits for response<br>direct turn (DT): it's your turn<br>Responds/gives feedback*<br>multimodal feedback (aFB): you are right, the monster feels happy<br>non-verbal feedback (anvFB): looks at the child, smiles, nods<br>gives answer (aAns): they feel disappointed because…<br>Encourages participation without requiring it*<br>personal comment (aPC): I think the child feels ashamed because he is naked [includes comments that respond to child's questions].<br>Encouraging comments (aEnC): you can do it; try it; wanna say something more?<br>Encourage participation using AAC<br>encourages AAC use (aEnAAC): you can use your [AAC system]
	Behaviors that may difficult or interfere in the child's participation	Inform about erroneous answer (ERAns)<br>Responds immediately [without waiting] (RI)<br>Interrupts the child (AIC)<br>Related to AAC<br>Keep AAC out of the child's reach (aKOAAC)<br>Ignores child's response (aIGN)
	Behaviors that promotes language comprehension and language clarification.	Explains vocabulary (aExV): thrilled means feeling extremely excited.<br>Interprets/clarifies communication (aCIC): the child says "sad," adult comments "ooh you mean that the penguin is sad"
	Behaviors that model communication	AAC model (aAAC): model the AAC use (select words in the child's system)<br>Gestures/Signs<br>manual signs model (aMS): "love" with manual signs.<br>emotional gesture model (aEG): surprised face<br>Pointing (aBP): point to the book or other object (no AAC) to give explanations/ comments. e.g., look at his face (pointing to the book) he is sad.<br>Recast (aRec): responses that correct or add grammatical detail and/or information to the child's utterance, e.g., child says "me angry," adult responds "I am angry"<br>Direct Communication Support (aDSC): adult supports the child directly to communicate (e.g., take child's hand to select the word in AAC, prompt to say a specific word or help physically to make a sign/gesture.)
	Behaviors that focus the child on tasks to perform	Orders/requests about tasks/behaviors to be carried out by the child<br>Positive order (PO): pay attention, manual sign of silence, come here.<br>Negative order (NO): don't do that, don't guess<br>Informative comments about the activity/task about to perform (ICT): we are going to read a book.<br>Physical support [excludes support to communicate]<br>To do something (TDS): helps the child turn the page.<br>To stop something (TSS): removes objects from the child's hand to stop distraction.

	Conversational content	Non-emotional content (AO): where is the flower? Is it red or blue? do you want the toys? This is like the one your grandma has, which one would you like?<br>Emotional content*<br>label (AECn): how do you feel? / she is scared<br>reason (AECca): why he feels sad? / she feels sad because…<br>responses/coping strategies (AECrs): what can he do? / he needs a hug.<br>unspecific label (AECun): he feels good/bad, I'm ok.
	Conversational theme* (related to)	Related to<br>storybook character (aCS): the dragon feels…, the grandma is so funny.<br>child (aCC): how would you [the child] feel? what would you do? What was your favorite part?<br>another person/situation (aCO): I [the mother] would do… I think your father will prefer to take a break.
	Behaviors related to the reading*	Reads (AR)<br>Explains the storybook (aCexB)<br>Paraphrase (P)
Child's interactive communication	Methods of expression*	Augmentative and Alternative Communication (cAAC)<br>Auditory perceptible<br>vocal (cVB)<br>speech (cS)<br>word approximation (cWA)<br>Visually perceptible<br>body movement (cBM): move head, change posture, etc.<br>action (cACT): run, jump, take something.<br>manual signs (cMS): sign language.<br>pointing (cP)<br>conventional gestures (cCG): gestures defined by the child's context with the intention to communicate (e.g., look up for yes, look down for no).<br>facial expressions (cFE): smiles.<br>emotional gestures (cEG): lips down for sad, crossing arms to express anger.
	Reasons to communicate	Refuses (cAv)<br>Obtains/solicits (cOb)<br>Behaviors in social interactions<br>Express interest/attracts attention (ISI)<br>Shows affection (ISA)<br>Shares (IS): share a toy.<br>Answers/gives information (cAnsw)<br>Asks questions (cQue)<br>Comments (cComm)<br>Asks for help/support (ISH)
	Conversational emotion-related content	Non-emotional content (COC)<br>Emotional content*<br>label (CECn): angry, sad, happy, excited.<br>reason (CECca): the witch doesn't have friends; the girl receives a present.<br>responses/coping strategies (CECrs): the little prince needs to express his feelings.<br>unspecific label (CECun): good, bad, ok, well.
	Conversational theme* (related to)	Related to*<br>storybook character (cCS): the penguin felt angry.<br>child (cCC): I would feel happy; I don't like that book.<br>another person/situation (cCO): my friend would feel ashamed.
Distractions*		Child distraction (CDI)<br>Adult distraction (ADI)
Impossibility of observation*		About the child<br>Lack of total visual access (cLTVA)<br>Lack of partial visual access (cLPVA)<br>Lack of auditory access (cLAA)<br>About the communication partner<br>Lack of total visual access (aLTVA)<br>Lack of partial visual access (aLPVA)<br>Lack of auditory access (aLAA)
Interruptions*		Ambiental activity interruption (AmI): noisy sound, phone call.<br>Video-camera interruption (VCI)

*category systems
